# Neoadjuvant immunotherapy versus chemoimmunotherapy in non-small cell lung cancer: A protocol for systematic review and meta-analysis

**DOI:** 10.1097/MD.0000000000033166

**Published:** 2023-03-03

**Authors:** Qunying Zhu, Guini Chen, Yunzhong Liu, Yu Zhou

**Affiliations:** a Department of Cardiothoracic Surgery, the First Affiliated Hospital of Hainan Medical University, Hainan, China; b Department of General Surgery, the First Affiliated Hospital of Hainan Medical University, Hainan, China.

**Keywords:** chemoimmunotherapy, meta-analysis, neoadjuvant immunotherapy, non-small cell lung cancer

## Abstract

Worldwide, lung cancer is the most common cause of cancer morbidity and mortality. Non-small cell lung cancer (NSCLC) accounts for approximately 80 to 85% of all lung cancers. Recently, a few studies have reported the use of neoadjuvant immunotherapy or chemoimmunotherapy in NSCLC. However, no meta-analysis comparing neoadjuvant immunotherapy with chemoimmunotherapy has yet been reported. We perform a protocol for systematic review and meta-analysis to compare the efficacy and safety of neoadjuvant immunotherapy and chemoimmunotherapy in NSCLC.

**Methods:**

The statement of preferred reporting items for systematic review and meta-analysis protocols will be used as guidelines for reporting the present review protocol. Original clinical randomized controlled trials assessing the beneficial effects and safety of neoadjuvant immunotherapy and chemoimmunotherapy in NSCLC will be included. Databases searched include China National Knowledge Infrastructure, Chinese Scientific Journals Database, Wanfang Database, China Biological Medicine Database, PubMed, EMBASE Database, and Cochrane Central Register of Controlled Trials. Cochrane Collaboration’s tool is used to assess the risk of bias in included randomized controlled trials. All calculations are carried out with Stata 11.0 (The Cochrane Collaboration, Oxford, UK).

**Results:**

The results of this systematic review and meta-analysis will be publicly available and published in a peer-reviewed journal.

**Conclusion:**

This evidence will be useful to practitioners, patients, and health policy-makers regarding the use of neoadjuvant chemoimmunotherapy in NSCLC.

## 1. Introduction

Worldwide, lung cancer is the most common cause of cancer morbidity and mortality. Approximately 247,270 new cases of lung cancer are estimated to occur in 2020, with 130,340 male cases and 116,930 female cases.^[1]^ Prior studies have reported that lung cancer resulted in more deaths than breast cancer, prostate cancer, colorectal cancer, and leukemia combined in men ≥ 40 years old and women ≥ 60 years old. With the introduction of screening guidelines and decrease in tobacco use, the mortality rate for lung cancer has recently decreased by 48% in males and 23% in females. Despite this decrease in mortality rate, approximately 140,730 deaths are estimated to be secondary to lung cancer in 2020.

The greatest risk factor for the development of lung cancer is tobacco use. Secondhand smoking has also been shown to increase the risk of lung cancer by as much as 26%. Other risk factors for lung cancer include asbestos exposure, family history of lung cancer, and exposure to toxic substances including polycyclic aromatic hydrocarbons, heavy metals, and radon gas.^[2]^

Non-small cell lung cancer (NSCLC) accounts for approximately 80 to 85% of all lung cancers^[3–6]^; its treatment depends on tumor histology, genetic subtype, performance status of the patient, and disease stage. Until now, surgery has been the cornerstone; nevertheless, around 40% of completely resected patients develop disease recurrence.^[7,8]^ There is growing enthusiasm for neoadjuvant immunotherapy in patients with resectable NSCLC.^[9,10]^ The proposed benefits of immunotherapy prescribed in the neoadjuvant setting include the increased release of neoantigens from the tumor to stimulate the expansion of specific T-cells, enhanced control of micro-metastases, and enabling the assessment of biologic and immunologic responses of the tumor from resected specimens.^[11]^ However, no meta-analysis comparing neoadjuvant immunotherapy with chemoimmunotherapy has yet been reported. We perform a protocol for systematic review and meta-analysis to compare the efficacy and safety of neoadjuvant immunotherapy and chemoimmunotherapy in NSCLC.

## 2. Methods

This review protocol has been registered in the International Prospective Register of systematic reviews. Its registration number was CRD42023395544. The statement of preferred reporting items for systematic review and meta-analysis protocols^[12]^ will be used as guidelines for reporting the present review protocol. This study comes from published data, so no ethical approval is required.

### 2.1. Inclusion criteria

Types of studies: Original clinical randomized controlled trials (RCTs) assessing the beneficial effects and safety of neoadjuvant immunotherapy and chemoimmunotherapy in NSCLC will be included. There will be no restrictions on publication language or publication status. Types of participants: Patients with resectable stages I to III NSCLC which was histologically confirmed in the tissue will be included. There will be no restrictions on the gender, age, or race of the participants.

Types of interventions: Intervention group receives preoperative neoadjuvant chemoimmunotherapy, while the control group receives neoadjuvant immunotherapy.

Types of outcomes: The primary outcomes are the overall long-term and disease-free survival outcomes. Perioperative mortality, surgical morbidity, and treatment-related adverse events are measured as secondary endpoints.

### 2.2. Database search strategy

Computer retrieval and manual retrieval will be used to retrieve all the published literature independently by 2 authors. Databases searched include China National Knowledge Infrastructure, Chinese Scientific Journals Database, Wanfang Database, China Biological Medicine Database, PubMed, EMBASE Database, and Cochrane Central Register of Controlled Trials. All relevant RCTs will be collected from the inception of each database to January 2023. The specific search strategy will be formulated with the specific database. We will search the reference lists of the relevant articles and will manually search Google Scholar to identify additional gray literature for inclusion. Table 1 shows the detailed search strategy in PubMed.

**Table 1:** Search strategy for the PubMed database.

#1	Non-small cell lung cancer [Title/Abstract]
#2	lung neoplasms [Title/Abstract]
#3	lung cancer [Title/Abstract]
#4	pulmonary neoplasm [Title/Abstract]
#5	NSCLC [Title/Abstract]
#6	#1 OR #2 OR #3 OR #4 OR #5
#7	neoadjuvant immunotherapy [Title/Abstract]
#8	neoadjuvant immune checkpoint inhibitor [Title/Abstract]
#9	neoadjuvant chemoimmunotherapy [Title/Abstract]
#10	nivolumab [Title/Abstract]
#11	durvalumab [Title/Abstract]
#12	atezolizumab [Title/Abstract]
#13	avelumab [Title/Abstract]
#14	ipilimumab [Title/Abstract]
#15	pembrolizumab [Title/Abstract]
#16	sintilimab [Title/Abstract]
#17	#7 OR #8 OR #9 OR #10 OR #11 OR #12 OR #13 OR #14 OR #15 OR #16
#18	randomly [Title/Abstract]
#19	randomized [Title/Abstract]
#20	RCT [Title/Abstract]
#21	#18 OR #19 OR #20
#22	#6 AND #17 AND #21

### 2.3. Study selection

**Figure 1.**
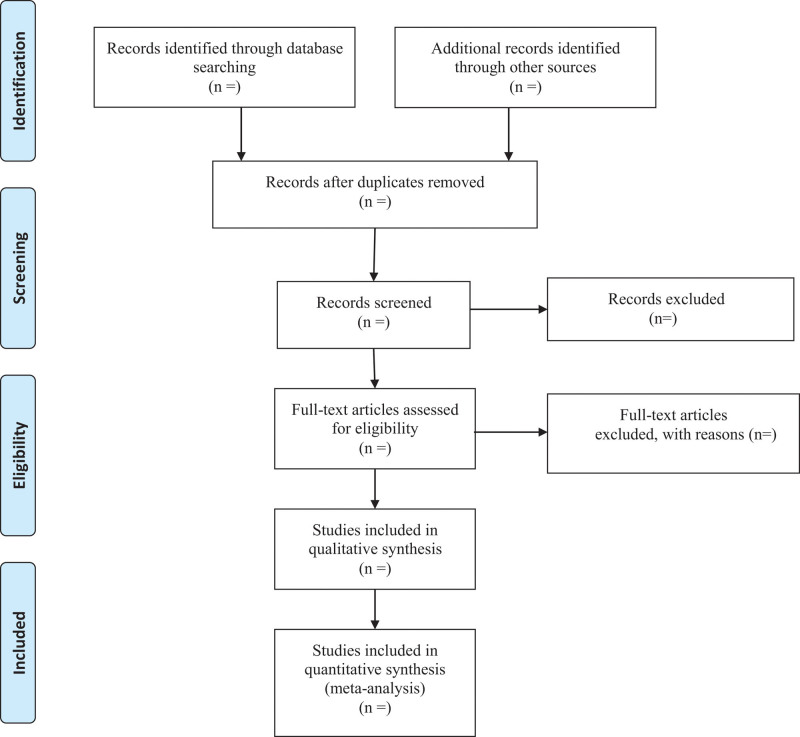
Flow diagram.

First, 2 researchers will screen independently to identify titles and/or abstracts of studies that potentially meet the inclusion criteria. Second, those 2 researchers will independently assess the full texts of these potentially eligible studies for eligibility. Any disagreements between them will be resolved through discussion between them. The procedures of study selection will be performed in accordance with the Preferred Reporting Items for Systematic reviews and Meta-Analysis flowcharts (as shown in Figure 1).

### 2.4. Data extraction

A standardized, pre-defined, pilot-tested form will then be used to extract data from the included studies for assessment of study quality and evidence synthesis. The extracted information will include the first author’s name, year of publication, country, sample size, and dropout, details of participants, experimental intervention, comparison, duration of intervention, main outcome measures, adverse events, and information for the assessment of the risk of bias (RoB). Two researchers will extract the data independently, and any discrepancies will be identified and resolved through discussion. When the data are insufficient, ambiguous, or missing, we will contact the corresponding authors of the original studies via e-mail.

### 2.5. Quality assessment

We will use the Cochrane Collaboration’s tool to assess the RoB of included RCTs.^[13]^ Domains include random sequence generation, allocation concealment, blinding of participants, personnel, and outcome assessors, completeness of data outcome, selective reporting, and other biases. In case of disagreement, the third investigator was responsible for resolving it.

### 2.6. Statistical methods

All calculations will be carried out with Stata 11.0 (The Cochrane Collaboration, Oxford, UK). Statistical heterogeneity is assessed based on the value of *P* and *I*^2^ using the standard chi-square test. When *I*^2^ > 50%, *P* < .1 is considered to be significantly heterogeneous. The random-effect model is performed for meta-analysis; otherwise, the fixed-effect model is used. When possible, subgroup analyses are conducted to explore the origins of the heterogeneity. The results of dichotomous outcomes are expressed as risk differences with a 95% confidence interval. For continuous various outcomes, mean difference or standardized mean difference with a 95% confidence interval is applied. Sensitivity analyses to identify the robustness of the results of the meta-analysis will be conducted by excluding studies with high RoB and outliers that are numerically distant from the rest of the data.

### 2.7. Assessment of reporting biases

If there are >10 studies included in the meta-analysis, a funnel plot will be used to assess publication bias.

## 3. Discussion

Lung cancer is the world’s leading cause of cancer death.^[14]^ Despite a trend towards an escalating diagnosis of resectable NSCLC, overall survival in patients with resectable NSCLC remains poor.^[15,16]^ The incorporation of chemotherapy into the neoadjuvant setting has improved disease-free survival, time-to-distant recurrence, and overall survival.^[17]^ Furthermore, the combination of chemotherapy and immunotherapy has improved pathological responses, which seem to be associated with increased survival.^[18]^ Although neoadjuvant immunotherapy may have benefits, its mechanism of action raises safety concerns when planning surgical resection. It remains unclear whether the inflammatory responses and immune-related adverse events that occur with neoadjuvant immunotherapy can present technical challenges and potentially compromise a planned resection. Our systematic review will provide a detailed summary of the current evidence related to the efficacy and safety of neoadjuvant chemoimmunotherapy in NSCLC. This evidence will be useful to practitioners, patients, and health policy-makers regarding the use of chemoimmunotherapy in NSCLC.

## Author contributions

**Conceptualization:** Guini Chen.

Data curation: Guini Chen.

Methodology: Yunzhong Liu.

Writing – original draft: Qunying Zhu.

Writing – review & editing: Yu Zhou.
